# Preferentially Expressed Antigen in Melanoma (PRAME) and Human Malignant Melanoma: A Retrospective Study

**DOI:** 10.3390/genes13030545

**Published:** 2022-03-19

**Authors:** Gerardo Cazzato, Katia Mangialardi, Giovanni Falcicchio, Anna Colagrande, Giuseppe Ingravallo, Francesca Arezzo, Giovanna Giliberti, Irma Trilli, Vera Loizzi, Teresa Lettini, Sara Scarcella, Tiziana Annese, Paola Parente, Carmelo Lupo, Nadia Casatta, Eugenio Maiorano, Gennaro Cormio, Leonardo Resta, Domenico Ribatti

**Affiliations:** 1 Section of Pathology, Department of Emergency and Organ Transplantation (DETO), University of Bari “Aldo Moro”, 70124 Bari, Italy; katia.mangialardi@gmail.com (K.M.); anna.colagrande@gmail.com (A.C.); giuseppe.ingravallo@uniba.it (G.I.); giovanna.giliberti@gmail.com (G.G.); teresa.lettini@uniba.it (T.L.); sara.scarcella@policlinico.ba.it (S.S.); eugenio.maiorano@uniba.it (E.M.); leonardo.resta@uniba.it (L.R.); 2 Section of Gynecology and Obstetrics, Department of Biomedical Sciences and Human Oncology (DIMO), University of Bari “Aldo Moro”, 70124 Bari, Italy; dr.falcicchio@gmail.com (G.F.); francesca.arezzo@uniba.it (F.A.); vera.loizzi@uniba.it (V.L.); gennaro.cormio@uniba.it (G.C.); 3 Odontostomatologic Clinic, Department of Innovative Technologies in Medicine and Dentistry, University of Chieti “G. d’Annunzio”, 66100 Chieti, Italy; trilliirma@gmail.com; 4 Department of Medicine and Surgery, LUM University, 70124 Casamassima, Italy; tiziana.annese@uniba.it; 5 Section of Human Anatomy and Histology, Department of Basic Medical Sciences, Neurosciences and Sensory Organs, University of Bari Medical School, 70124 Bari, Italy; domenico.ribatti@uniba.it; 6 Pathology Unit, Fondazione IRCCS Casa Sollievo della Sofferenza, 71013 San Giovanni Rotondo, Italy; paolaparente77@gmail.com; 7 Innovation Department, Diapath S.p.A, Via Savoldini n.71, 24057 Martinengo, Italy; carmelo.lupo@diapath.com (C.L.); nadia.casatta@diapath.com (N.C.)

**Keywords:** PRAME, malignant melanoma, skin, differential diagnosis, histopathology

## Abstract

Background: Preferentially expressed antigen in melanoma (PRAME) is a cancer testis antigen (CTA) identified in 1997 through analysis of the specificity of tumor-reactive T-cell clones derived from a patient with metastatic cutaneous melanoma. Although at first it seemed even more specific, various studies have shown that PRAME can also be expressed in the context of atypical lesions that do not correspond solely to the definition of malignant melanoma. Methods: A systematic review of English articles was conducted following the Preferred Reporting Items for Systematic Reviews and Meta-Analyses (PRISMA) guidelines. Results: 126 records were identified in the literature search, of which 9 were duplicates. After screening for eligibility and inclusion criteria, 53 publications were included. Conclusions: The advent of a new marker such as PRAME is surely a step forward not only in the diagnostic approach, but also in the immunotherapeutic approach to MM. However, various studies have shown that PRAME can also be expressed in the context of atypical lesions apart from MM and, for this reason, the diagnostic sensitivity and specificity (hence accuracy) are clearly lower. Further studies with larger case series will be necessary to understand better what possibilities are offered in terms of diagnostic reliability by PRAME.

## 1. Introduction

Despite advances in therapy and treatment, malignant melanoma (MM) continues to be a very aggressive skin cancer, with 324,635 new cases and 57,043 deaths worldwide in 2020, accounting for 1.7% of all cancers in the world population [1], and showing a rising incidence [2]. Histological diagnosis continues to be of great importance in the diagnostic and therapeutic care pathway of patients suffering from malignant melanoma [3] and although it is often relatively simple, at other times, the recognition of the malignant lesion can pose a great challenge [4]. For this reason, alongside conventional histopathology, ancillary immunohistochemistry techniques have been introduced, in an attempt to simplify the difficulties of diagnosis [5]. Despite this, researchers have always tried to find new markers that could help, particularly in challenging cases of MM [6], such as Melan-A (MART-1) [7], HMB-45 (anti-human melanosome clone HMB45) [8], and MITF (melanocyte inducing transcription factor) [9], but the morphological difficulty together with the negativity of some histotypes of MM (such as desmoplastic melanoma) [10], has meant that even these have failed to completely resolve the diagnostic dilemmas. The widespread use of Sry-related HMg-Box gene 10 (SOX-10) has made it possible to solve cases of differential diagnostics (such as differential diagnoses between some histotypes of MM and other types of skin lesions) [11,12], but a certain degree of difficulty still remains. PRAME (preferentially expressed antigen in melanoma) is a cancer testis antigen (CTA) that was first identified in 1997 through analysis of the specificity of tumor-reactive T-cell clones derived from a patient with metastatic cutaneous melanoma [13]. Over the years, however, it was found that PRAME was not only expressed in cutaneous melanoma, but also in other types of MM including ocular, as well as in various malignant neoplasms not of melanocyte origin, such as non-small cell lung cancer [14], breast cancer [15], renal cancer [16], ovarian cancer [17], hematological malignancies [18,19,20], synovial sarcoma, and myxoid liposarcoma [21,22]. Healthy tissues do not express PRAME except the testis, ovary, placenta, adrenal glands, and endometrium [23]. In this paper, we focus on the state of the art regarding the diagnostic utility of PRAME in immunohistochemistry for the histopathological diagnosis of MM, make a careful review that also includes the other applications of this CTA, and, finally, envisage possible future developments.

## 2. Materials and Methods

A systematic review was conducted following the Preferred Reporting Items for Systematic Reviews and Meta-Analyses (PRISMA) guidelines. A search of PubMed, MEDLINE, and Web of Sciences (WoS) databases was performed until 22 February 2022 using the terms: preferentially expressed antigen in melanoma (PRAME) in combination with each of the following: melanoma, neoplasm, and immunohistochemistry. Only articles in English were selected. Eligible articles were assessed according to the Oxford Centre for Evidence-Based Medicine 2011 guidelines [24]. Review articles, meta-analyses, observational studies, and letters to the editor were included. Other potentially relevant articles were identified by manually checking the references of the included literature.

An independent extraction of articles was performed by two investigators (G.C. and A.C.) according to the inclusion criteria. Disagreement was resolved by discussion between the two review authors. Because the study designs, participants, treatment measures, and reported outcomes varied markedly, we focused on describing the different approaches of the authors regarding the immunoexpression of PRAME in malignant melanoma, analyzing the techniques (mainly immunohistochemistry) used in the works examined. Finally, we analyzed the state of the art, imagining what the future perspectives may be.

## 3. Results

**Figure 1:**
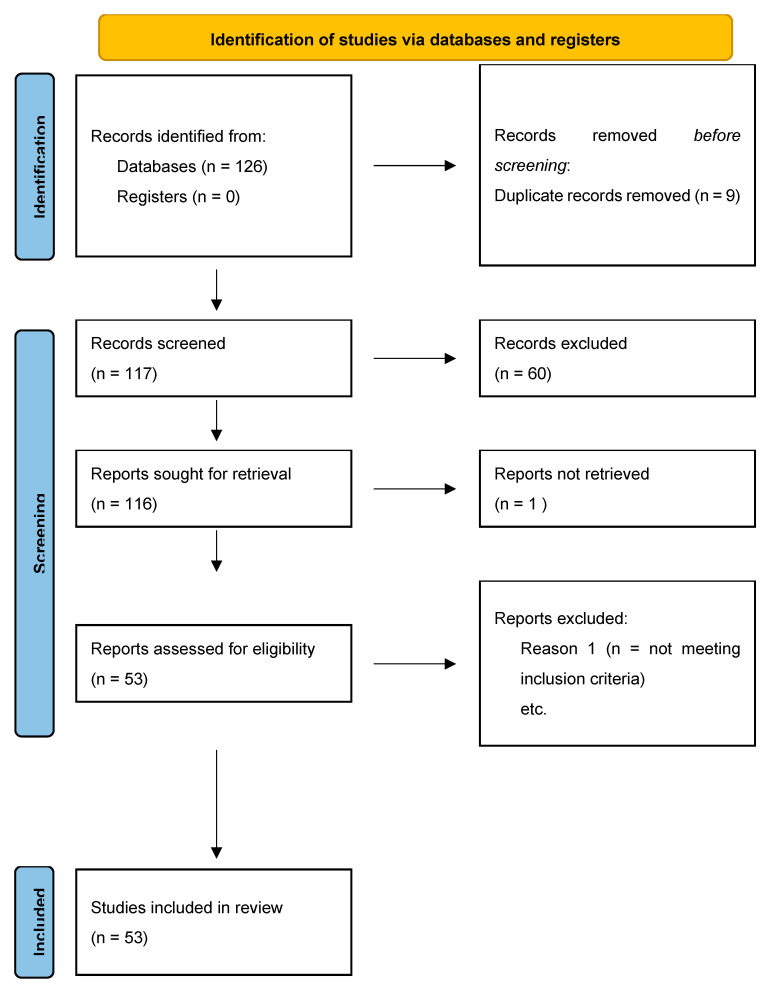
PRISMA flowchart used in review about PRAME.

In total, 126 records were initially identified in the literature search, of which nine were duplicates. After screening for eligibility and inclusion criteria, 53 publications were ultimately included (Figure 1). Most of the publications were original or research articles, or both (n = 12), followed by reviews with or without metanalysis (n = 2), a comparative study (n = 1), and a case-control retrospective study (n = 1). All studies included were rated as level 4 or 5 for evidence for clinical research as detailed in the Oxford Centre for Evidence-Based Medicine 2011 guidelines [24].

## 4. Discussion

Since its first description back in 1997 by Ideka et al. [13], PRAME has been of interest both as a possible diagnostic parameter and as a target for possible immunotherapy. Since then, various studies have tried to shed light on the real potential use of this marker, and in recent years the aspect relating to immunoexpression has been particularly studied in the context of differential histopathological diagnostics of MM [25,26,27]. Recent works by Lezcano C. et al. [25,26,28] have clarified some important aspects relating to the role of PRAME as a diagnostic aid. In fact, in one of the first studies conducted on this topic, the authors [25] presented their findings regarding the immunoexpression of PRAME in 400 melanocytic tumors, including 155 primary and 100 metastatic melanomas, and 145 melanocytic nevi. Diffuse nuclear immunoreactivity for PRAME was found in 87% of metastatic and 83.2% of primary melanomas. Among melanoma subtypes, PRAME was diffusely expressed in 94.4% of acral melanomas, 92.5% of superficial spreading melanomas, 90% of nodular melanomas, 88.6% of lentigo maligna melanomas, and 35% of desmoplastic melanomas. When in situ and nondesmoplastic invasive melanoma components were present, PRAME expression was seen in both. Of the 140 cutaneous melanocytic nevi, 86.4% were completely negative for PRAME. Immunoreactivity for PRAME was seen, albeit usually only in a minor subpopulation of lesional melanocytes, in 13.6% of cutaneous nevi, including dysplastic nevi, common acquired nevi, traumatized and recurrent nevi, and Spitz nevi (one case in a 6 years-old child). Rare isolated junctional melanocytes with immunoreactivity for PRAME were also seen in solar lentigines and benign non lesional skin. In this first work, it was highlighted that this marker could certainly constitute a valid support in the differential diagnosis of benign and malignant pigmented lesions, albeit with limitations in selected cases. In a 2020 paper, Lezcano et al. [26,28] reported a series of cases of immunostaining for PRAME: they examined 45 nodal melanocytic deposits comprising 30 nodal nevi and 15 melanoma metastases. All nodal nevi (30/30) were negative for PRAME, whereas all melanoma metastases (15/15) were diffusely positive for PRAME IHC. Furthermore, the authors reported the utility of PRAME/Melan A dual-label immunostaining and stressed the usefulness of PRAME IHC in the assessment of diagnostically challenging nodal melanocytic deposits, such as intraparenchymal nodal nevi, metastases confined to the capsular fibrous tissue, or in the setting of small metastases coexisting with a nodal nevus in the same lymph node. These aspects were also analyzed by See et al. [29]. In a paper published in September 2020, Gradecki S. et al. reported their experience of immunostaining of PRAME on 155 cases of metastatic melanoma, 54 of which were to lymph node and 101 to non-lymph node sites. PRAME expression was seen in 151/155 (97.4%) cases, with 4+ expression in 64 cases (41.3%), 3+ expression in 46 cases (29.7%), 2+ expression in 18 cases (11.6%), and 1+ expression in 23 cases (14.8%). Lymph node metastases were more likely to show a lower expression as compared to metastases to other anatomic sites. Based on these data, the authors suggested the possibility that PRAME could be of diagnostic aid in confirming a diagnosis of MM in a metastatic setting (both lymph node and other sites) [30].

Considering that in the biology of malignant transformation from dysplastic nevus to MM a series of genetic-molecular events occur, that alter the expression of various protein molecules [31,32], Lohman et al. [33] investigated the immunoexpression pattern of PRAME in melanomas associated with a dysplastic nevus (NAM). In that paper they reviewed thirty-six cases: 67% (24/36) of melanomas were PRAME positive (4+) while no (0/36) nevi showed 4+ positivity; 81% (29/36) of nevi were completely PRAME negative compared to 17% (6/36) of melanomas. In 67% of cases (24/36) PRAME differentiated between benign and malignant melanocyte populations. The authors identified a high rate (67%) of differential PRAME staining in adjacent benign and malignant melanocyte populations in NAM. In PRAME positive (4+) melanomas, PRAME differentiates 100% (24/24) of benign and malignant melanocyte populations. When 4+ staining is used as the threshold for positivity, PRAME staining has a sensitivity of 67% (24/36) and a specificity of 100% (36/36). These results supported the concept that PRAME IHC can assist in distinguishing melanocyte populations in melanoma arising within nevi.

A study by Raghavan et al. investigated further and confirmed the usefulness of PRAME IHC for making differential diagnoses between melanocytic proliferations with intermediate histopathology and melanoma: they found that traumatized, mitotically-active, persistent and recurrent, and dysplastic nevi usually lacked PRAME expression altogether. As in the study by Lezcano et al. [26], the need for further histopathological, cytogenetic, and molecular characteristics to interpret the PRAME status in cases of spitzoid neoplasia was confirmed. Indeed, although most benign and intermediate Spitz lesions lacked widespread PRAME expression, widespread PRAME positivity was observed in a Spitz nevus and atypical Spitz tumor. Furthermore, immunoreactivity intensity ranged from weak to strong, regardless of the degree of atypia [34].

Scherlfer et al. conducted a study on the relationship between clinical features, gene expression profile (GEP) class, and PRAME expression in uveal melanoma. There was no association between PRAME expression and clinical features (gender, patient age, and tumor thickness). PRAME staining was not statistically associated with a higher TNM stage. However, it should be noted that the GEP class was associated with higher TNM staging and worsening of the GEP class was associated with positive PRAME status. PRAME expression was found to be associated with an increased risk of metastasis and a worse prognosis in all GEP classes.

PRAME expression was associated with the largest basal diameter (LBD) and tumor volume. Notwithstanding, PRAME expression can appear at any stage of tumor progression and not only in advanced stages. These findings were in agreement with the already known strong association of LBD with GEP and metastatic risk [35]. These data have also since been confirmed and expanded on in the paper by Cai L. et al [36].

In a cases series reported by Hovander et al. PRAME expression was studied in eight cases of oral malignant melanoma (OMM), a rare and very aggressive neoplasm with a high risk of metastasis. In this study, PRAME was positive in 83.2% of the OMMs analyzed [37]. In a 2021 paper, Jue hu et al. [38] studied the expression pattern of PRAME in acral lentiginous melanoma (LM) and acral nevi (ANs) that had never been previously analyzed; 89.3% of ALMs resulted PRAME positive, and 94.1% of ANs resulted in being completely PRAME negative. The PRAME expression proportion of tumor cells in the epidermidis was slightly higher than in the dermis. The PRAME positive proportion of epithelioid cells was slightly higher than that of spindle cells; 82.6% of ALMs with lymph node involvement at diagnosis expressed PRAME, compared with 57.6% of those without. PRAME has both a good sensitivity (69.3%) and high specificity (100%) for discriminating ALMs from ANs.

In this scenario, rising attention is being devoted to this marker, and new studies are being conducted in an attempt to increase experiences, report results, and find safer answers [39,40,41,42,43,44,45,46,47,48,49,50,51,52,53,54,55,56,57,58,59].

In an elegant paper published in 2021, Lopez et al. described their pilot study on PRAME immunostaining of 24 lesions that are particularly difficult to diagnose: 24 lesions consisting of five cases of low-grade inactivated melanocytic tumor (BIMTs), seven cases of deep penetrating nevus (DPNs), and 12 combined nevi with conventional and DPN features (CDPNs). A total of five BIMTs were analyzed in regard to PRAME, and none had an immunoreactivity score greater than 1+, while all the BIMT cases scored higher than zero demonstrated a weak staining intensity. Of the remaining 19 cases, seven were DPNs and 12 were CDPNs. None of the DPN/CDPN cases demonstrated an immunoreactivity score greater than 2+, except for one CDPN. Of the DPN/CDPN cases scored higher than zero, only two demonstrated strong intensity, neither of which showed salient distinguishing morphologic features. None of the 24 cases examined demonstrated diffuse positivity (score: 4+). This work offered the authors a slightly greater confidence in the possibility of PRAME providing a practical diagnostic aid in the course of the diagnostic management of these particular lesions [60].

In 2020, Leczano et al. demonstrated a high concordance between PRAME immunoexpression results and cytogenetic data, starting from ambiguous melanocytic lesions and difficult diagnostic categorization [61]. In this work, the authors analyzed 110 cases of particularly challenging melanocytic lesions and found a 90% agreement between the PRAME IHC and the cytogenetic test results (fluorescence in situ hybridization or single nucleotide polymorphism-array, or both) and a concordance of 92.7% between PRAME IHC and the final diagnosis. However, it was clearly accepted that IHC and cytogenetics were not interchangeable, as there is the possibility of false negative or false positive results [61].

Very recently, in 2022, Krajisnik et al. published a paper where they evaluated the expression of the PRAME protein in a series of melanocytic lesions of the nail. In their work, 25 nail unit melanomas (including small biopsy and amputation samples) and 32 control benign melanocytic lesions were retrospectively reviewed. PRAME nuclear staining was evaluated (similarly to other works in the literature) as a percentage and intensity labeling. All melanoma cases showed PRAME nuclear expression, which was usually widespread and strong. In samples with few cancer cells, staining was restricted to tumor cells, corresponding to the initial H&E imprint. All control cases were negative for PRAME expression. The expression of PRAME was useful in distinguishing between melanomas and other melanocytic lesions of the nail. This antibody has also been shown to be diagnostically valuable for detecting melanoma cells in small samples with minimal disease [62].

## 5. Conclusions

PRAME expression in melanoma cells is regulated by many actors. Hypermethylation appears to downregulate the expression of PRAME [63], while downregulation of miR-211 (an RNA gene) results in increased expression [64]. Aberrant PRAME hypomethylation results in an augmented transcription and a higher risk of metastasis in uveal melanoma. [65] The activity of MZF-1 also increases the expression of PRAME in melanoma cells and their ability to form colonies [63,66]. A retrospective study conducted on different types of mucosal melanoma (sinonasal, gastrointestinal, genitourinary, and oropharyngeal) shows the prognostic implication of PRAME expression. PRAME expression was lower in surviving patients at 24 months, and higher PRAME staining detected on immunohistochemical analysis was correlated with a 170% risk of death [67]. Furthermore, mucosal melanomas analyzed by Toyama et al. [67] showed a higher PRAME expression in cells with an epithelioid than a spindle morphology.

From a molecular point of view, mucosal melanomas are more similar to uveal melanoma than to cutaneous melanoma [25,46,68].

PRAME is expressed by melanoma and many different tumors: a higher expression is typical of advanced diseases and is associated with lymph node spread of the disease. Prognosis is poor when PRAME is overexpressed, indicating worse disease-free, progression-free, and metastasis-free prognosis, and worse overall survival [69].

## Data Availability

Not applicable.
